# Ultrasound-guided endoscopy to improve accuracy of persistent urogenital sinus (PUGS) diagnosis in adult patient: A case report

**DOI:** 10.3389/fsurg.2023.1105551

**Published:** 2023-03-09

**Authors:** Mariateresa Mirandola, Benedetta Gui, Angelica Naldini, Nazario Foschi, Viola Casula, Antonia Carla Testa, Giovanni Scambia, Ursula Catena

**Affiliations:** ^1^ Department of Woman and Child Health, Fondazione Policlinico Universitario A. Gemelli IRCCS, Rome, Italy; ^2^ Department of Urology, Fondazione Policlinico Universitario Agostino Gemelli IRCCS, Rome, Italy; ^3^ Department of Woman and Child Health, Università Cattolica del Sacro Cuore, Rome, Italy

**Keywords:** endoscopy, hysteroscopy, ultrasound, mullerian malformation, congenital malformation, PUGS-persistent urogenital sinus

## Abstract

**Introduction:**

persistent urogenital sinus (PUGS) is a rare condition characterized by abnormal communication between the urethra and vagina, that can frequently be associated with other complex Mullerian malformation (33%). We present a case of PUGS associated with a complex Mullerian malformation diagnosed in adult age after the integration of gynecological ultrasound with hysteroscopy, both performed by expert operators.

**Case description:**

27-year-old women was referred to our clinic because of frequent urinary tract infections and cyclic pelvic pain. She was virgo, with normal menstrual cycles and dysmenorrhea. A didelphys uterus and double vagina with bilateral hematocolpos was firstly diagnosed through transrectal and transabdominal ultrasound. An MRI was then performed and a monorenal and ipsilateral ureteral malformation were diagnosed; in addition, a complete absence of the lower third of the vagina and an abnormal origin of the urethra from the bladder were described. Patient underwent lower genital tract endoscopy: external vaginal orifice was obliterated, a PUGS was diagnosed and both vaginas' ostia were detected on the PUGS's posterior-lateral walls. The procedure was performed under transabdominal ultrasound guidance which confirmed the endoscopic anatomical suspicion, avoiding complications such as perforation and misdiagnosis.

**Discussion:**

ultrasound guided endoscopy plays an essential role in the evaluation of complex anatomic anomalies, such as persistent urogenital sinus (PUGS), leading to a dynamic one-stop diagnosis; it avoids delays and misdiagnosis in preoperative assessment possibly related to the separately application of different radiological and endoscopic techniques.

## Introduction

Persistent urogenital sinus (PUGS) is a rare condition characterized by abnormal confluence of the female genital anatomy in the urinary tract (1–5). PUGS is a variant of persistent cloaca, a rare malformation occurring in 2,8:100.000 (3) where an abnormal confluence of the urinary tract, the female genital tract and the ano-rectal tract coexist in a single cavity communicating externally. Patients affected by PUGS don't have normal vagina nor a normal urethra. PUGS is a small tubulated structure, greater in diameter and length compared to normal urethra, connecting the external introit with the bladder; it is covered by squamous epithelial lining, like vagina's epithelium. It can frequently be associated with renal or ano-rectal malformations or other complex Mullerian malformations (33%) (4–8). In these patients, vagina usually ends posteriorly or laterally in the PUGS. The incidence of congenital genitals anomalies in general population is estimated to be 0.001%–10% (9), and they can be associated with infertility and recurrent miscarriages or can be asymptomatic. Congenital genital malformations are usually diagnosed occasionally after routine gynecological reports or during infertility insight, and surgery is not always indicated (10).

Unlike Mullerian genital malformations, PUGS are typically diagnosed in pediatrics' age, due to the urinary tract symptoms, and immediately surgically corrected because of the short- and long-term consequence that may be associated (11).

Correct preoperative evaluation is crucial for adequate surgical planning. Reported PUGS had been diagnosed with a combination of MRI-magnetic resonance imaging- and cystoscopy, requiring multiple diagnostic steps and a multidisciplinary approach (12, 13). We present a case of PUGS associated with a complex Mullerian malformation misdiagnosed in pediatrics age and correctly framed and described in adult age, after the integration of gynecological ultrasound with hysteroscopy both performed by expert operators. The case report description was performed following the CARE-Case Report Guidelines criteria (14).

## Case description

**Figure 1:**
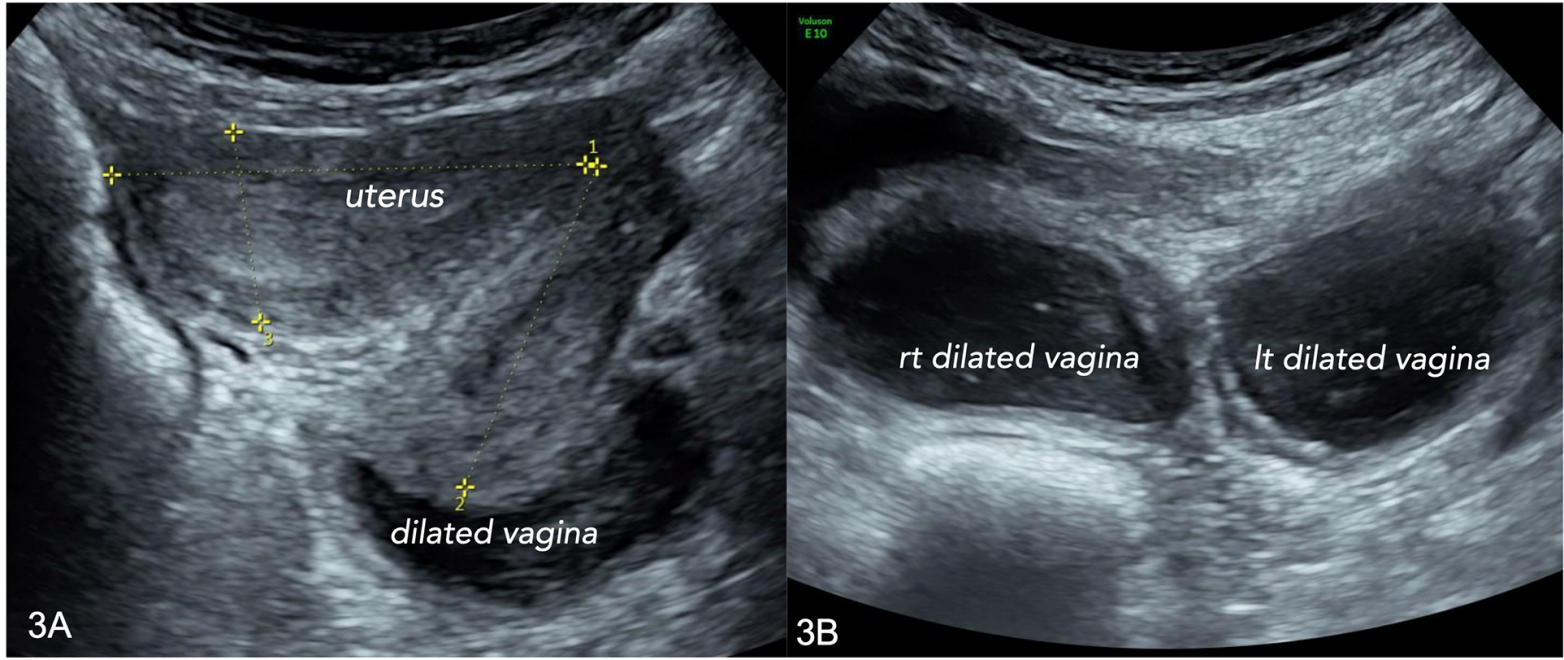
Transabdominal ultrasound. (**A**) right uterus and vagina, longitudinal scan; (**B**) both vaginas, dilated aspect, with “low-level” content, in in a transverse scan.

27-year-old women was referred to our clinic because of frequent urinary tract infections and cyclic pelvic pain. She had had previous neonatal surgery because of unperforated anus. No family history of congenital uro-genital malformation was reported. She was virgo, with normal menstrual cycles, dysmenorrhea, and frequent urinary tract infections during menses. At the gynecological department of Policlinico A. Gemelli, Rome, Italy, a didelphys uterus and double vaginas with bilateral hematocolpos were diagnosed through transrectal and transabdominal ultrasound (Figure 1). A Mullerian malformation classified as Complete Bicorporeal Uterus, Double Cervix, Longitudinal obstructing vaginal septum [U3b C2 V2 according to ESHRE/ESGE classification (15)] was firstly diagnosed. An MRI was then performed to deepen the study of the complex Mullerian malformation: a left renal malformation and an ipsilateral ureter malformation were diagnosed; in addition, a complete absence of the lower third of the vagina and an abnormal origin of the urethra from the bladder were described. In order to understand how she could possibly have normal menstrual cycles with a vaginal atresia, patient underwent ultrasound-guided lower genital tract endoscopy in our Digital Hysteroscopy Clinic where ultrasound can be easily integrated in endoscopic approaches (1).

## Diagnostic assessment

**Figure 2:**
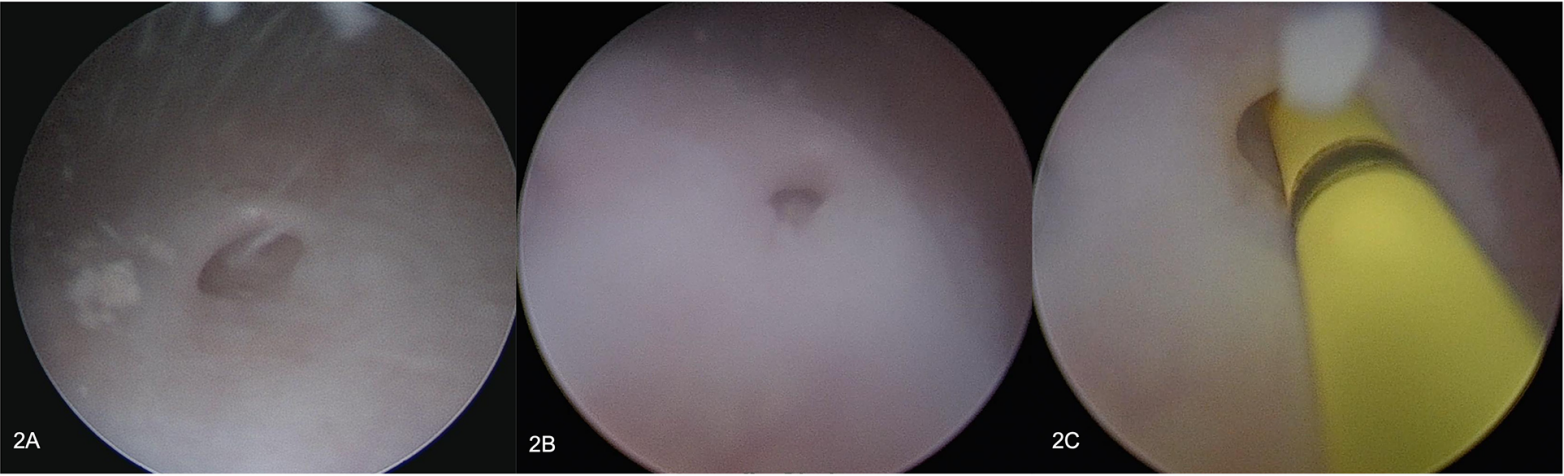
Endoscopy, ureters. (**A**) left ureter; (**B**) right ureter; (**C**) cannulated left ureter.

**Figure 3:**
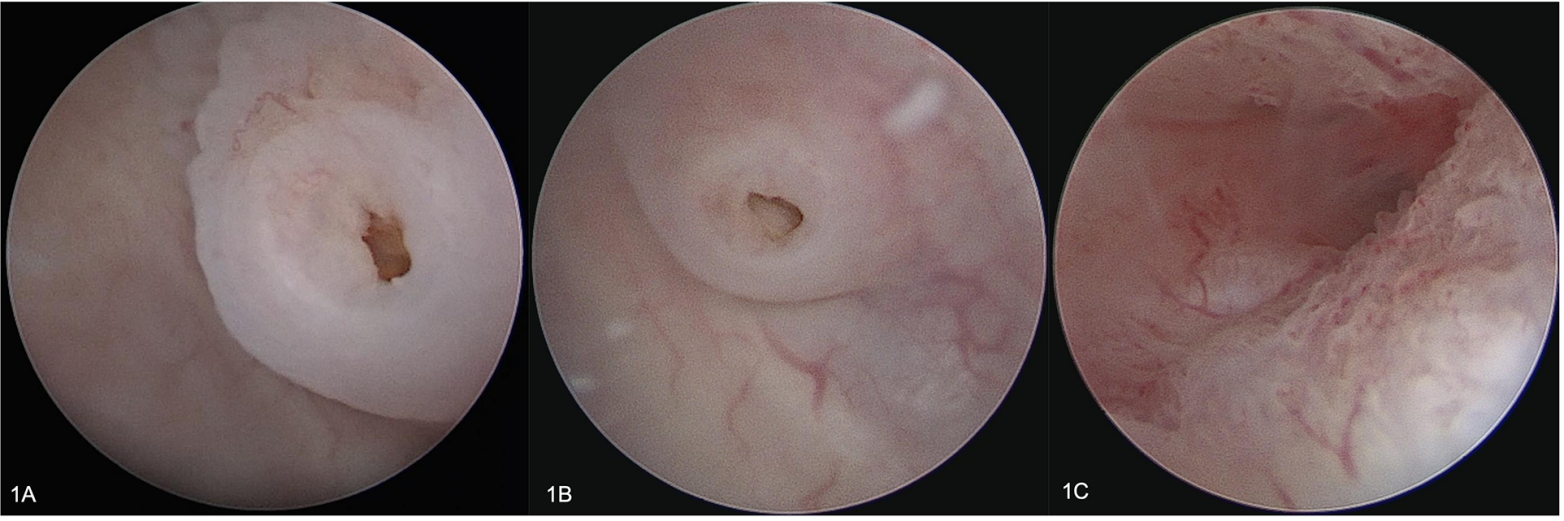
Endoscopy. (**A**) left vaginal ostia on the PUGS lateral-posterior wall; (**B**) right vaginal ostia on the PUGS on the lateral posterior-wall; (**C**) left endocervix.

**Figure 4:**
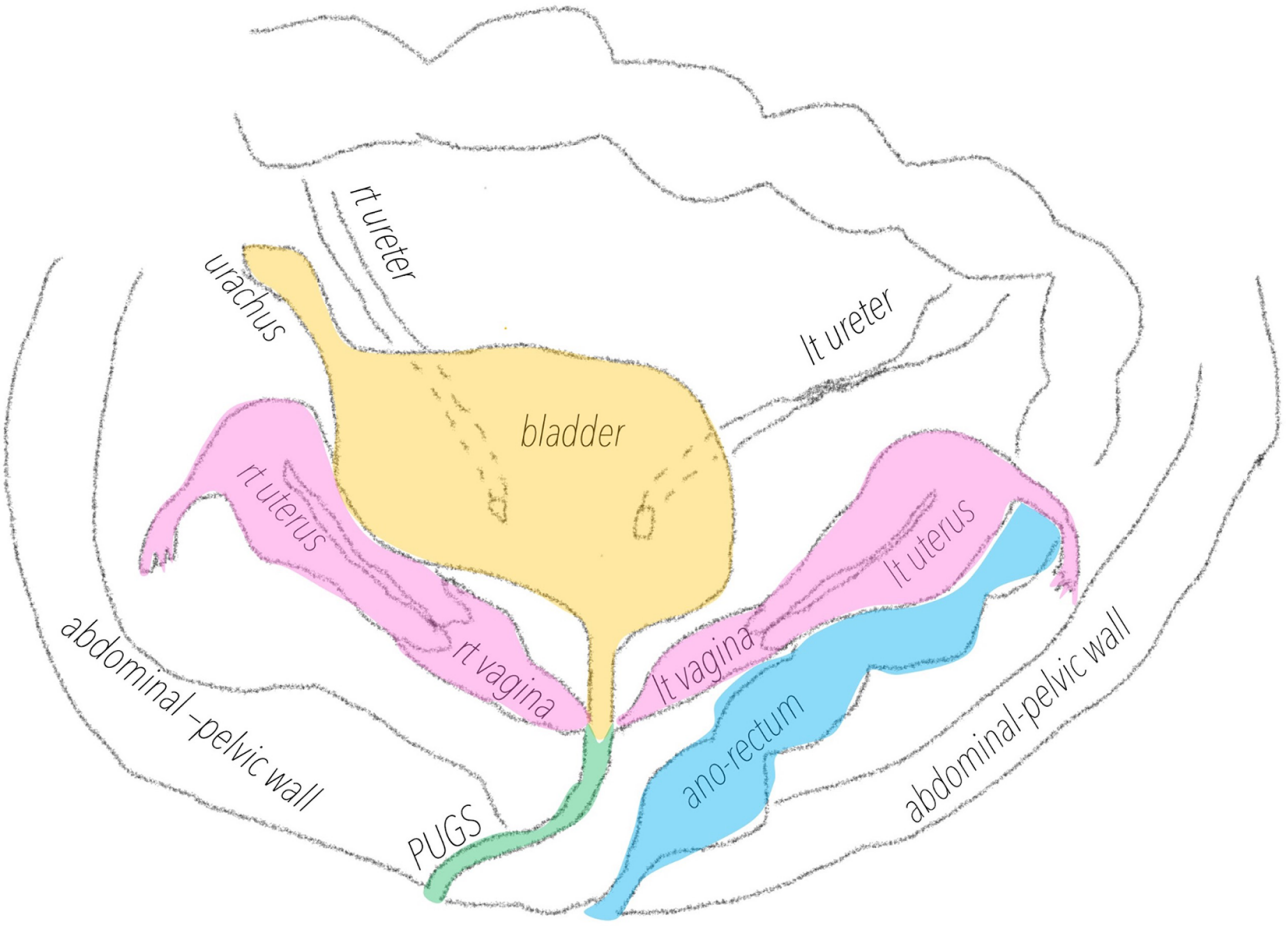
Schematic representation of PUGS and the associated Mullerian malformation.

The external vaginal orifice was obliterated. A 5 mm hysteroscope with continuous flow was inserted through a misdiagnosed persistent urogenital sinus (PUGS), covered by squamous epithelial lining. PUGS was 7 cm long and ended in a dilated bladder that was full of blood clots and dense fluid. Multiple washings were performed until both ureteral ostia have been identified. Moreover, a dead-end residual urachus was diagnosed. The right ureter was cannulated and described as normal; the left ureter could only be cannulated for a few centimeters after entering in the bladder, demonstrating the presence of a complete obstruction (Figure 2). Exiting from the bladder, the surgeon deeply analyzed the persistent urogenital sinus walls and identified the presence of two millimetric ostia. To avoid false paths, the hysteroscope was carefully inserted in both ostia under transabdominal ultrasound guidance, entering two separate vaginas; both cervical canals were identified. Vaginas' walls were dilated and covered by squamous epithelial lining like normal vagina epithelium. Cervices were normal and both cervical canals were covered by endocervical glandular epithelial lining (Figures 3, 4). The procedure was performed under transabdominal ultrasound guidance which confirmed endoscopic anatomy, avoiding complications such as perforation and misdiagnosis.

## Discussion

The diagnosis of PUGS associated with a complex Mullerian malformation was completed thanks to the integration of ultrasound and hysteroscopy performed by expert operators. In this case, PUGS was misdiagnosed for years, and an incomplete surgical intervention had been performed in childhood. The gynecological ultrasound examination in adult age has revealed the presence of a complex Mullerian malformation and the subsequent MRI diagnosed the urinary tract malformations. The final dynamic integration of lower genital tract endoscopy and trans-abdominal ultrasound led to correct diagnosis.

The strength of our reported case is the description of the achievement of correct diagnosis through the integrated technique of ultrasound guided performance of lower genital tract endoscopy in an adult woman affected by complex congenital uro-genital malformation. Limitations may be represented by the few literature data regarding this complex malformation and the single-case description.

Complex congenital uro-genital malformations often represent a challenge for the gynecologist and the urologist to be diagnosed. The achievement of correct diagnosis usually requires multiple steps and techniques, different specialist collaborations and integration of multiple radiological and endoscopic techniques. Time to achieve correct diagnosis is often long, misdiagnosis are frequent and premature surgical interventions may be hazardous. The described integrated approach, ultrasound combined with lower uro-genital tract endoscopy, allows to completely and correctly evaluate the suspected complex Mullerian malformation previously studied by MRI.

In conclusion, ultrasound-guided endoscopy plays an essential role in the evaluation of complex anatomic anomalies such as persistent urogenital sinus (PUG), leading to correct preoperative diagnosis. Suspected complex congenital uro-genital malformations should be evaluated by expert operators; we believe specialized center should integrate ultrasound and endoscopy in a dynamic diagnostic step in order not to delay diagnosis or to avoid misdiagnosis and unproper surgical interventions.

## Data Availability

The original contributions presented in the study are included in the article/Supplementary Material, further inquiries can be directed to the corresponding author/s.
